# Vaginal temperature as indicative of thermoregulatory response in Nellore heifers under different microclimatic conditions

**DOI:** 10.1371/journal.pone.0223190

**Published:** 2019-10-01

**Authors:** Caroline Carvalho de Oliveira, Fabiana Villa Alves, Paulo Gustavo Macedo de Almeida Martins, Nivaldo Karvatte Junior, Geovani Ferreira Alves, Roberto Giolo de Almeida, Ariadne Pegoraro Mastelaro, Eliane Vianna da Costa e Silva

**Affiliations:** 1 Faculty of Veterinary Medicine and Animal Science, Federal University of Mato Grosso do Sul, Campo Grande, MS, Brazil; 2 Embrapa Beef Cattle, Campo Grande, MS, Brazil; 3 Department of Animal Science, Federal University of Jequitinhonha and Mucuri Valleys, Diamantina, MG, Brazil; 4 Faculty of Veterinary and Animal Science, Federal University of Goiás, Goiânia, GO, Brazil; 5 Faculty of Engineering, Architecture and Urbanism, and Geography, Federal University of Mato Grosso do Sul, Campo Grande, MS, Brazil; 6 Faculty of Animal Science, Federal University of Paraná, Paraná, PR, Brazil; University of Illinois, UNITED STATES

## Abstract

The objective was to evaluate vaginal temperature as a thermoregulatory response of Nellore heifers under different microclimatic conditions. The study was conducted during one year in an experimental area located at 54°37′W, 20°27′S, and 530 m of altitude in Brazil. Twenty-four Nellore females were reared in integrated crop-livestock-forestry systems with different shading patterns. The randomized complete block experimental design was utilized with a sub subdivided plots scheme, with plot corresponding to the production systems, the subplot to the season of the year, and the sub subplot, the hours of the day. To characterize the microclimate, data regarding air and black globe temperatures, and air relative humidity were collected and from them, temperature-humidity index was calculated. Vaginal temperature was obtained by using a bottom-type temperature logger (i-button) attached to an adapted intravaginal device. The black-globe temperature explained the variation on vaginal temperature. Increases on vaginal temperature were only observed, on average, two hours after the increase of black-globe temperature. The microclimatic conditions found in the systems, resulting from the various tree densities, modify the vaginal temperature in different degrees, demonstrating that the shading effect is not always accompanied by improvements in thermal comfort. The system with intermediate density showed a better microclimatic condition and, consequently, a lower increase in vaginal temperature. The interaction between air temperature, humidity and solar radiation resulted in adverse environmental conditions, however, Nellore heifers showed good adaptation to the environment. In conclusion, vaginal temperature is a good indicator to evaluate the thermoregulatory response in Nellore heifers.

## Introduction

Currently, one of the limitations found in tropical beef cattle systems is the negative effect of heat stress on the productive indexes. Bovines are considered homeothermic animals for the ability to maintain, almost constant, their internal temperature, through mechanisms of loss or gain of body heat, even in the case of environmental variations within the acceptable limits of the animal organism [1]. Heat tolerance in cattle has been increasingly used as a selection tool for adaptation to more inhospitable regions. However, several factors are involved in the determination of thermal comfort and acclimation to specific environments. Among them, there are factors related to the environment, which involve climatic elements, and factors related to the animal, such as the physiological responses associated, mainly, with the thermoregulatory mechanism [2].

Attempts to establish criteria for classification of animals less or more adapted to particular environments, as well as to characterize the environment according to their thermal comfort condition, have been increasingly explored, since there are differences in the intensity of responses to adversities between species, breeds, and even individuals. The evaluation of an animal's ability to respond through a climatic condition has been based on the following physiological variables: (1) rectal temperature; (2) respiratory frequency; and (3) heart rate [3]. Under heat stress conditions, especially in regions that combine high temperatures and relative air humidity, these variables are able to indicate the efficiency of the animal in losing the acquired heat [4]. Together, indexes related with climatic elements have also been used as a tool to evaluate the environmental condition in which the animal is reared [1].

Recent studies in cattle have shown vaginal temperature as an efficient physiological response to environmental variation. Although several studies show a close relationship between rectal and vaginal temperatures, little is known about its variation in cattle, especially in the Nellore breed, considered to be one of the most adapted to the tropical climate, especially because of its relation with climatic adversities. Thus, our objective was to evaluate vaginal temperature as a thermoregulatory response of Nellore heifers under different microclimatic conditions.

## Materials and methods

### Location

**Fig 1:**
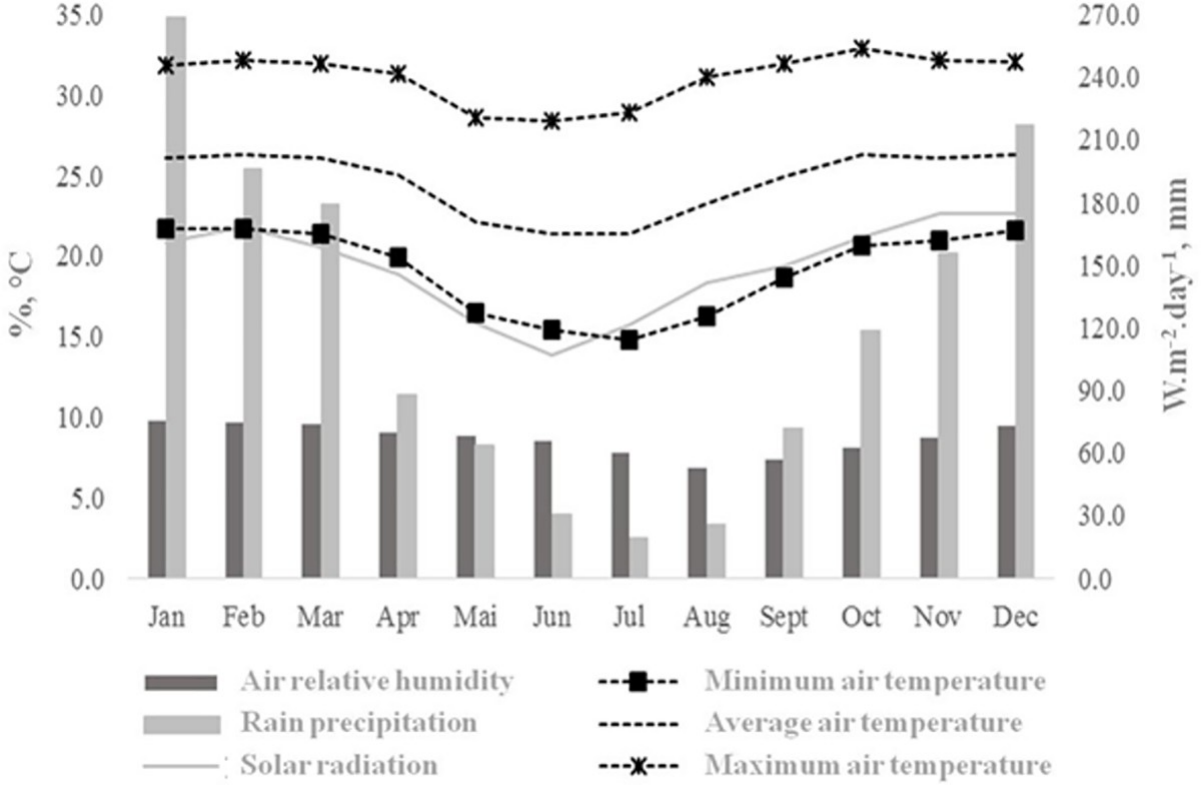
Provisional climatic norms of the region where the experiment was realized, in Mato Grosso do Sul State, Brazil, between 1992 and 2013 (adapted from Flumignan et al. [8]).

The present study was conducted during one year at the Embrapa Beef Cattle, in Campo Grande, Mato Grosso do Sul State, Brazil (54°37′W, 20°27′S, and 530 m of altitude). This location is geographically localized at the central portion of the state (which is located in the mid-west region of the country) in the neotropis zone of the biogeography region of savannah [5]. According to the Köppen-Geiger climate classification [6], the experimental area is located in the transition between Cfa and AW tropical humid, characterized by a regular rain distribution, with an average annual precipitation of 1560 mm, dry season occurring during the coldest months (May to September) and rainy season during the summer (October to March), with an average annual temperature of 23°C [7]. Standardized climatological normals are depicted in Fig 1.

### Experimental area

The experimental site was previously described by Oliveira et al. [9, 10]. In brief, the 18-ha experimental area consisted of integrated crop-livestock-forestry (ICLF) systems and was established in 2008 as a strategy of pasture recuperation by cultivating soybeans followed by Piatã-grass (*Urochloa brizantha* cv. BRS Piatã) as the forage component, managed under continuous stocking rate system and variable number of animals.

**Fig 2:**
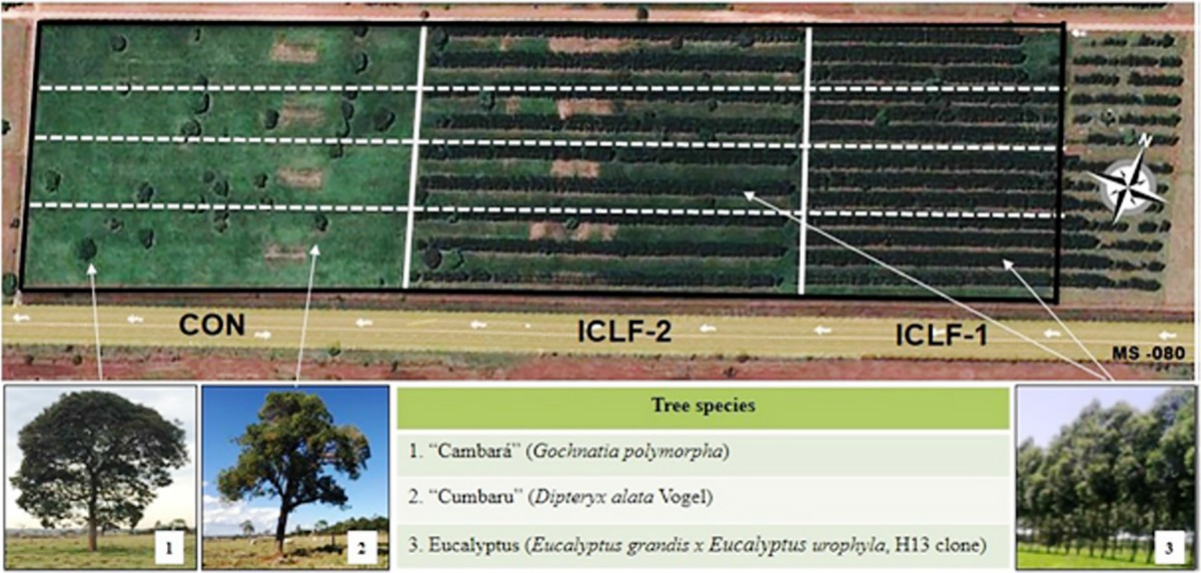
Experimental site and the representation of the tree species present in the integrated crop-livestock-forest (ICLF) systems (ICLF-1 and ICLF-2) and the control (CON) system.

Two ICLF systems (ICLF-1 and ICLF-2) were implanted with eucalyptus trees (*Eucalyptus grandis x Eucalyptus urophylla*; H13 clone; during the experimental period, initial average height was 26 m and the final, 30 m), disposed in a single row differing on tree spacing and density (14 x 2 m with 357 trees per ha, and 22 x 2 m with 227 trees per ha, respectively). Tree rows had a dislocation of -20.41° S and -54.71° W, relative to the east-west axis. An integrated crop-livestock system (control; CON) was utilized in study and was composed of five scattered native trees (*Gochnatia* and *Dipteryx* species) per hectare. The area was divided into 12 1.5-ha paddocks (four per system) with mineral and water troughs (Fig 2).

Animals were provided with diets to meet the nutrient requirements according to their breed and age. Commercial mineral supplement and clean fresh water were provided *ad libitum* during the experimental period. The nutritional value presented by the forage component in the experimental period corresponded to the organic matter content of 91.72, 91.73 and 91.66%, and crude protein of 10.18, 9.22 and 8.47% during the winter; during the summer, organic matter was 91.92, 91.67 and 91.69%, and crude protein, 9.87, 8.32 and 6.91%, in ILPF-1, ILPF-2 and CON, respectively.

### Animals, management, and experimental design

Twenty-four 16-month-old Nellore heifers, with average initial body weight of 264.8 kg, were utilized. Animals were treated against endo and ectoparasites according to the need of control of horn fly (*Haematobia irritans*) and ticks (*Rhipicephalus microplus*), throughout the experimental period. Animals were randomly distributed, with two heifers used to evaluate animal parameters, and other heifers with similar BW were used to keep predetermined sward heights. Animals utilized in this study were cared for by acceptable practices (considering the welfare ones) and the research procedures and methodologies were previously approved by the Institutional Committee of Ethics on the Use of Animals from the Federal University of Mato Grosso do Sul (protocol number 511/2013).

Heifer body weight (BW) was measured in 28-day intervals, after a 12-hour period of water and feed withdrawal. Total BW gain was measured by calculating the difference between initial and final BW (referring to the day when vaginal device used to measure vaginal temperature was inserted) and average daily gain (ADG) was calculated dividing total BW by 28 days of evaluation.

The randomized complete block experimental design was utilized in the present study in sub subdivided plots scheme. Treatments corresponding to production systems (ICLF-1, ICLF-2 and CON) were considered as the main plot; season of the year (winter and summer), the subplot; and hours of the day (1 to 24 h), the sub-subplot.

### Microclimatic parameters and thermal comfort indexes

Microclimatic data were collected in July (winter) and December (summer), coinciding with vaginal temperature measurement periods. These data were collected daily during 28 consecutive days, at one-hour intervals, starting at 00:00 (GMT-04: 00), totaling 24 records per day.

Air temperature, dew point temperature and air relative humidity were measured using a digital thermo-hygrometer (model HT-500; Instrutherm Instrumentos de Medição Ltda., São Paulo, Brazil), placed in meteorological shelters, as recommended by Trumbo et al. [11]. To measure black globe temperature, the thermo-hygrometers were encapsulated in round PVC shields (0.15 cm in diameter) painted in matte black, as proposed by Souza et al. [12].

**Fig 3:**
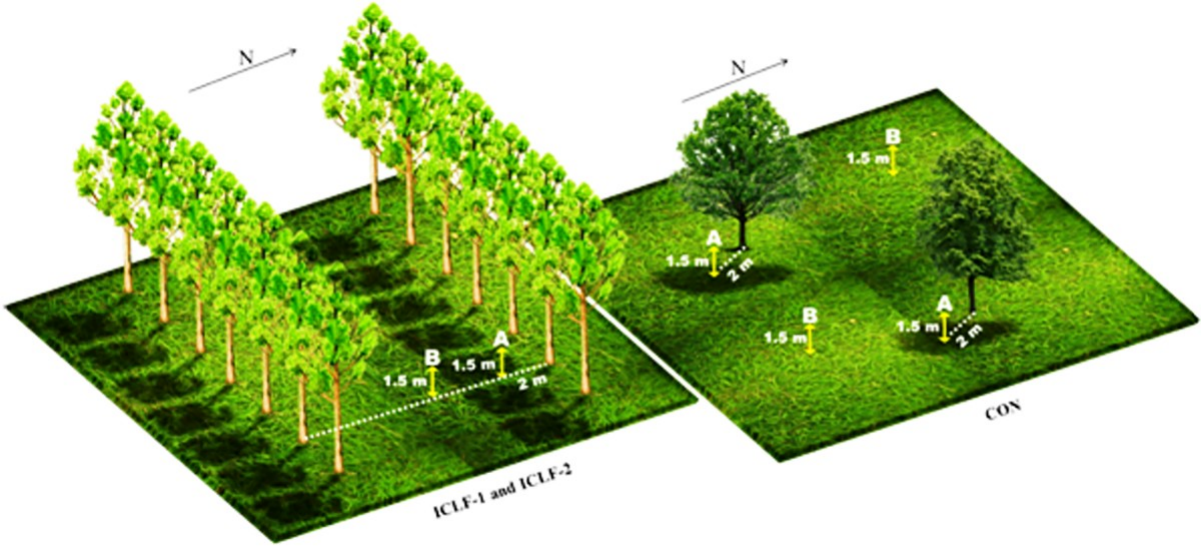
Schematic diagram of sampling points (A and B) in the integrated crop-livestock-forest (ICLF) systems (ICLF-1, with 357 trees per ha and ICLF-2, with 227 trees per ha), and the control (CON) system (with five remaining native trees per hectare).

During the day, the devices were placed in two different points: under the shade (A) and under the sun (B), at 1.5 m from the soil surface (which correspond to the center of mass of beef cattle), considering the variation of the shade projection and angle (Fig 3). During the night, the devices were placed 2 m from the trees (A) and in a central point (B). Two repetitions of each sampling point were utilized in each pasture. Meteorological data were collected daily by a meteorological station (A702–INMET) distant approximately 4 km from the experimental area.

From the microclimatic data, to evaluate animal thermal comfort condition, the black globe temperature and humidity index (BGHI) was calculated using the equation proposed by Buffington et al. [13]:

BGHI=bgt+0.36wbt+41.5 (1)

in which bgt is the black globe temperature and wbt is the wet-bulb temperature. The BGHI values, according to Baêta [14], were classed by the National Weather Service as follows: comfort (up to 74); alert (74 to 79); danger (79 to 84); and emergency (above 84).

### Vaginal temperature measurement

Vaginal temperature was measured by using a bottom-type temperature logger (iButton Temperature Loggers, model DS1922L, Maxim Integrated Products, Inc., San Jose, CA, United States). The temperature loggers utilized in the present study were brand new, purchased already calibrated according to the company parameters. To ensure the loggers were recording correctly the temperature, they were placed in an oven at 50°C for one hour and programmed to record the temperature in one-minute intervals. After this procedure, the registration pattern was verified. Since the temperature loggers were first-time use, the recorded temperatures were the same. The calibration standard of the company can be found at https://www.maximintegrated.com/en/app-notes/index.mvp/id/4629. The logger was inserted into a blank intravaginal device which was adapted to hold it as described by Burdick et al. [15]. The temperature sensor had 17.35 mm in circumference, 5.89 mm in diameter and 3.3 g, and, according to the manufacturer, the device has a temperature accuracy of ±0.5°C from -10°C to +65°C. Before the insertion of the temperature logger, the intravaginal devices were subjected to asepsis during two days, intercalating periods of soaking and washing, every six hours, with a cleaning laboratorial detergent (Extran MA 02 neutro, Merck S/A Brasil, São Paulo, Brazil), and then sterilized by autoclaving. The vaginal probes were kept in heifers for a total of 56 days. Temperature sampling and recording rate were set at one-hour intervals for 28 days in the months of July (winter) and December (summer) of 2015. The vaginal probe was inserted into the vagina with an applicator. After sampling period, it was removed and the data were transferred to a personal computer using the OneWireViewer software (version 1.3; Maxim Integrated Products, Inc.). No clinical inflammatory reaction or vaginal discharge was detected that may have affected the obtained values for vaginal temperature.

### Statistical analysis

Normal distribution of the data was checked through normality test using the UNIVARIATE procedure of SAS (version 9.2; SAS Inst., Inc., Cary, NC, USA). Thus, because we obtained quantitative data, with normal distribution and independence between observations, the Pearson correlation (r) was performed to identify and quantify linear relations from the correlation coefficients found between vaginal temperature, and microclimatic variables. Correlation coefficients were classed as: |r| = 0.00, null; 0.00 < |r| < 0.20, very weak; 0.20 < |r| < 0.40, weak; 0.40 < |r| < 0.60, moderate; 0.60 < |r| < 0.80, strong; |r| > 0.80, very strong; e |r| = 1.00, perfect. Then, analysis of variance was performed using the GLM procedure of SAS, considering system, season, hour of the day and all possible interactions as fixed effects, and initial BW as covariate. In addition, regression analysis was performed using the REG procedure of SAS, considering the statistical model:

$$Y_{ijkl}=\alpha +S_{i}+S_{ej}+H_{k}+\beta_{1}X_{1ijkl}+\beta_{2}X_{2ijkl}+\ldots +\beta_{p}X_{pijkl}+e_{ijkl}$$ (2)

in which: Y_ijkl_ is the dependable variable in the lth observation, performed in the ith system of the jth season in the kth hour; α is the intercept; S_i_ is the effect of the ith system; Se is the effect of the jth season; H_k_ is the effect of the kth hour; Β_m_ (m = 1, …, p) are the coefficients of partial regression on the environmental variables (independent variable) which measures are the X_mijkl_ values, corresponding to the Y_ijkl_ observation; and *e*_iijkl_ is the random error.

Average values of vaginal temperature, total BW gain, and black-globe temperature were separated for comparison by Tukey’s Studentized Test at *P* ≤ 0.05. In case of significant interactions, the averages were adjusted by using the LSMEANS statement of SAS. The statistical model utilized was:

$$Y_{ijkl}=\mu +B_{i}+S_{j}+e_{ij}+E_{k}+{SE}_{jk}+e_{ijk}+H_{l}+{SH}_{jl}+{EH}_{kl}+{SEH}_{jkl}+e_{ijkl}$$ (3)

in which: μ is the constant; B_i_ is the effect of the ith block, i = 1, …, 4; S_j_ is the effect of the jth system, j = 1, 2, 3; *e*_ij_ is the error a; E_k_ is the effect of the kth season, k = 1, 2; SE_jk_ is the effect of the jth system and kth season; *e*_ijk_ is the error b; H_l_ is the effect of the lth hour, l = 1, …, 24; SH_ji_ is the effect of the jth system in the lth hour; EH_kl_ is the effect of the kth season in the lth hour; SEH_jkl_ is the effect of the interaction between jth system, kth season and lth hour; and *e*_ijkl_ is the residue.

## Results

Raw data collected throughout the experiment are presented in S1 Dataset. During the winter, the lowest air temperature (23.8, 23.4, and 23.7°C), black globe temperature (25.2, 23.7, and 23.8°C), air relative humidity (59.9, 58.9, and 59.2%), and BGHI (72.0, 70.2, and 70.4) were recorded on ICLF-1, ICLF-2, and CON, respectively. During the summer, air temperature was 27.8, 27.8, and 27.4°C, black globe temperature was 28.4, 28.7, and 28.6°C, air relative humidity was 71.6, 70.8, and 72.7%, and BGHI was 77.7, 78.0, and 77.9 for ICLF-1, ICLF-2, and CON, respectively. Vaginal temperature presented weak positive correlation with season, hour of the day, total BW gain, and ADG (Table 1). Weak and positive correlations were also observed with air temperature and dew point temperature, and very weak and positive correlation with BGHI.

**Table 1:** Pearson correlation coefficients (r) between vaginal temperature from Nellore heifers with seasons, hours of the day, animal performance and climatic elements in systems with different microclimatic conditions.

Item	Vaginal temperature	
	r	*P*-value
System	0.0408	0.0009
Season	0.3413	< 0.0001
Hour of the day	0.3034	< 0.0001
Total body weight gain	0.3178	< 0.0001
Average daily gain	0.3215	< 0.0001
Air temperature	0.2014	< 0.0001
Dew point temperature	0.2814	< 0.0001
Black globe temperature	0.1306	0.5358
Relative humidity	0.0077	< 0.0001
Black globe temperature and humidity index	0.1689	< 0.0001

Total BW gain and ADG did not present correlation (*P* > 0.05) with system and hour of the day (Table 2). On the other hand, total BW gain and ADG were positively correlated and with a strong correlation with season (*P* < 0.0001). Weak and positive correlations were obtained for air temperature and black globe temperature with total BW gain and ADG. Dew point temperature presented positive correlation with animal performance (*P* < 0.0001), with moderate magnitude for total BW gain and strong for ADG. Weak and positive correlations were found between BGHI and total BW gain, and between BGHI and ADG. Data regarding partial regression coefficients and respective standard deviations of a linear function for the vaginal temperature of Nellore heifers in systems with different microclimatic conditions are described in Table 3.

**Table 2:** Correlation matrix of the variables systems, seasons, hour of the day, performance of Nellore heifers, and climatic elements of systems with different microclimatic conditions.

	Total BW gain	ADG	Air temp.	Dew point temp.	Black globe temp.	Relative humidity	BGHI
System	0.0206	0.0142	-0.0849*	-0.0342*	-0.1285*	0.0814*	-0.1191*
Season	0.7419*	0.8026*	0.4189*	0.7293*	0.3821*	0.1207*	0.4751*
Hour of the day	0.0001	0.0001	0.0676*	0.0170	0.0457*	-0.0850*	0.0433*
Total BW gain	1	0.9936*	0.3391*	0.5721*	0.2995*	0.0824*	0.3725*
ADG	-	1	0.3583*	0.6093*	0.3183*	0.0912*	0.3962*
Air temp.	-	-	1	0.5842*	0.9177*	-0.6871*	0.9165*
Dew point temp.	-	-	-	1	0.5789*	0.1694*	0.6996*
Black globe temp.	-	-	-	-	1	-0.5997*	0.9876*
Relative humidity	-	-	-	-	-	1	-0.4929*
BGHI	-	-	-	-	-	-	1

*Significant at *P* ≤ 0.05.

BW = body weight; ADG = average daily gain; BGHI = black globe temperature and humidity index

**Table 3:** Partial regression coefficients and their respective standard deviations of a linear function for the vaginal temperature of Nellore heifers in systems with different microclimatic conditions.

Item	B	Standard deviation	*P*-value
Intercept	34.0568	0.3904	< 0.0001
Season	0.7036	0.0453	< 0.0001
Hour of the day	0.0262	0.0009	< 0.0001
Total BW gain	0.0848	0.0097	< 0.0001
ADG	-0.0017	0.0002	< 0.0001
Initial BW	0.0049	0.0003	< 0.0001
Black globe temperature under the sun	-9.9283	3.2628	0.0024
Black globe temperature under the sun under the shade	-9.9356	3.2634	0.0023
Average black globe temperature	19.8223	6.5262	0.0024
Average BGHI	0.0350	0.0087	< 0.0001

ADG = average daily gain; BW = body weight; BGHI = black globe temperature and humidity index.

**Fig 4:**
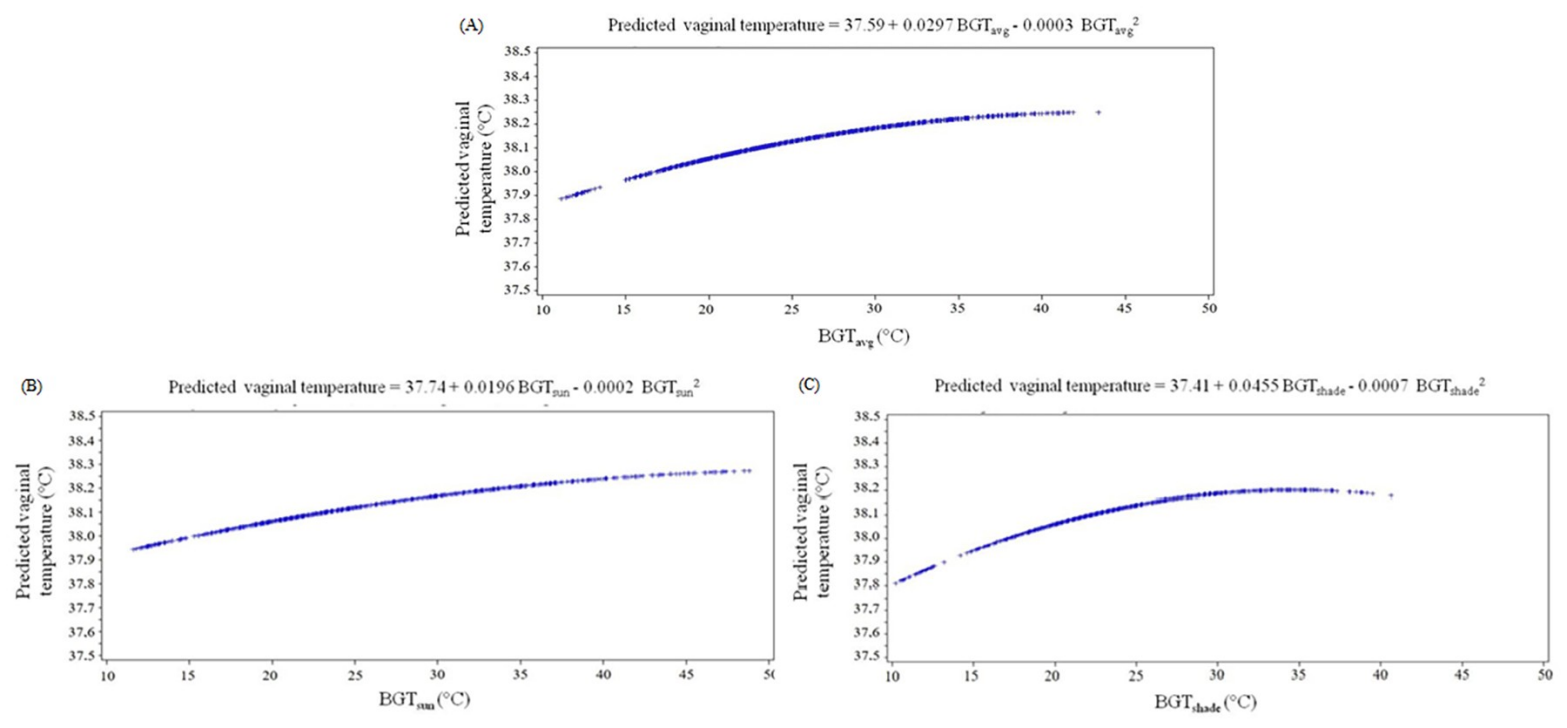
Vaginal temperature of Nellore heifers as a function of the average black globe temperature–BGT (A); BGT measured under the sun (B); and under the shade (C) of systems with different microclimatic conditions.

Variations in the average black globe temperature did not produce the same response pattern in vaginal temperature (Fig 4). For each unit of increase in the average black globe temperature between 0 and 8°C, an increase of 0.03°C was observed in vaginal temperature. Between 9 and 25°C, the increase in vaginal temperature was 0.02°C, and between 26 and 41°C, 0.01°C. For black globe temperature measured under the sun, between 0 and 12°C, an increase of 0.02°C was observed, whereas between 13 and 37°C, 0.01°C. On the other hand, for black globe temperature measured under the shade, an increase of 0.04°C in vaginal temperature was observed for each unit of increase between 1 and 7°C; between 8 and 15°C, the increase was 0.03°C, 0.02°C between 16 and 22°C, and 0.01°C between 23 to 29°C. Between 30 and 36°C, vaginal temperature was not altered. For black globe temperatures measured under the shade above 37°C, the effects on vaginal temperature are inverted: as one degree increases in black globe temperature measured under the shade, the vaginal temperature decreases up to -0.03°C.

**Fig 5:**
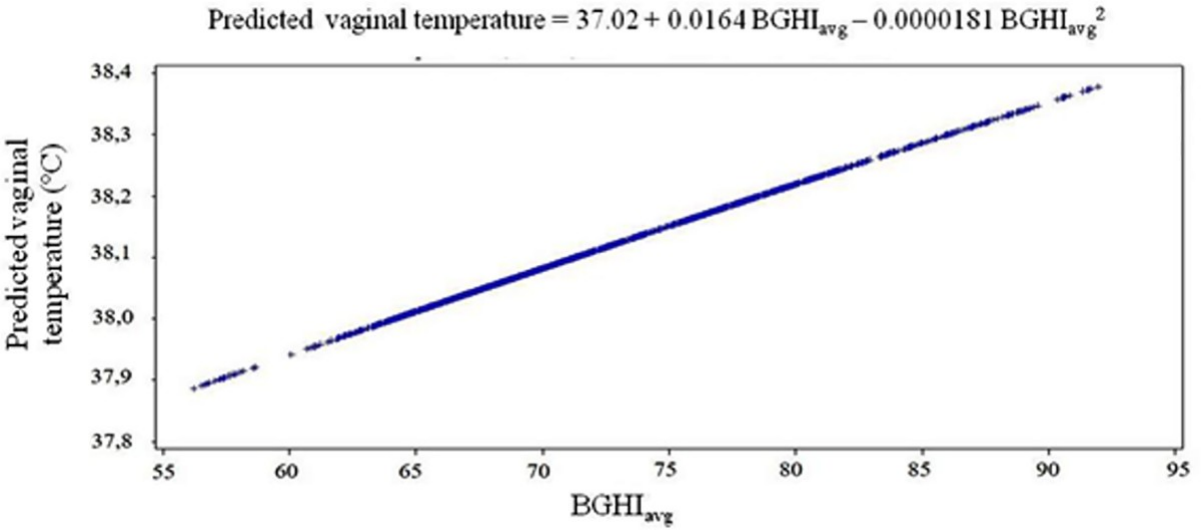
Vaginal temperature of Nellore heifers as a function of the average black globe temperature and humidity index (BGHI) of systems with different microclimatic conditions.

Vaginal temperature presented the same response pattern as the average BGHI increased. In a vaginal temperature of 37.02°C, the BGHI value was null. For each unit increase in BGHI, an increase of 0.0164°C in the vaginal temperature was observed (Fig 5).

During the winter, the average black globe temperature began to increase from 06:00, reaching the maximum value between 12:00 and 13:00, and decreasing from 14:00. During the summer, this temperature increased from 07:00, reaching the maximum value at 16:00 on ICLF-1 and ICLF-2, and at 13:00 on CON. Vaginal temperature started increasing at 08:00, reaching maximum value at 16:00 and 17:00 in the summer and winter, respectively, decreasing after these hours.

**Fig 6:**
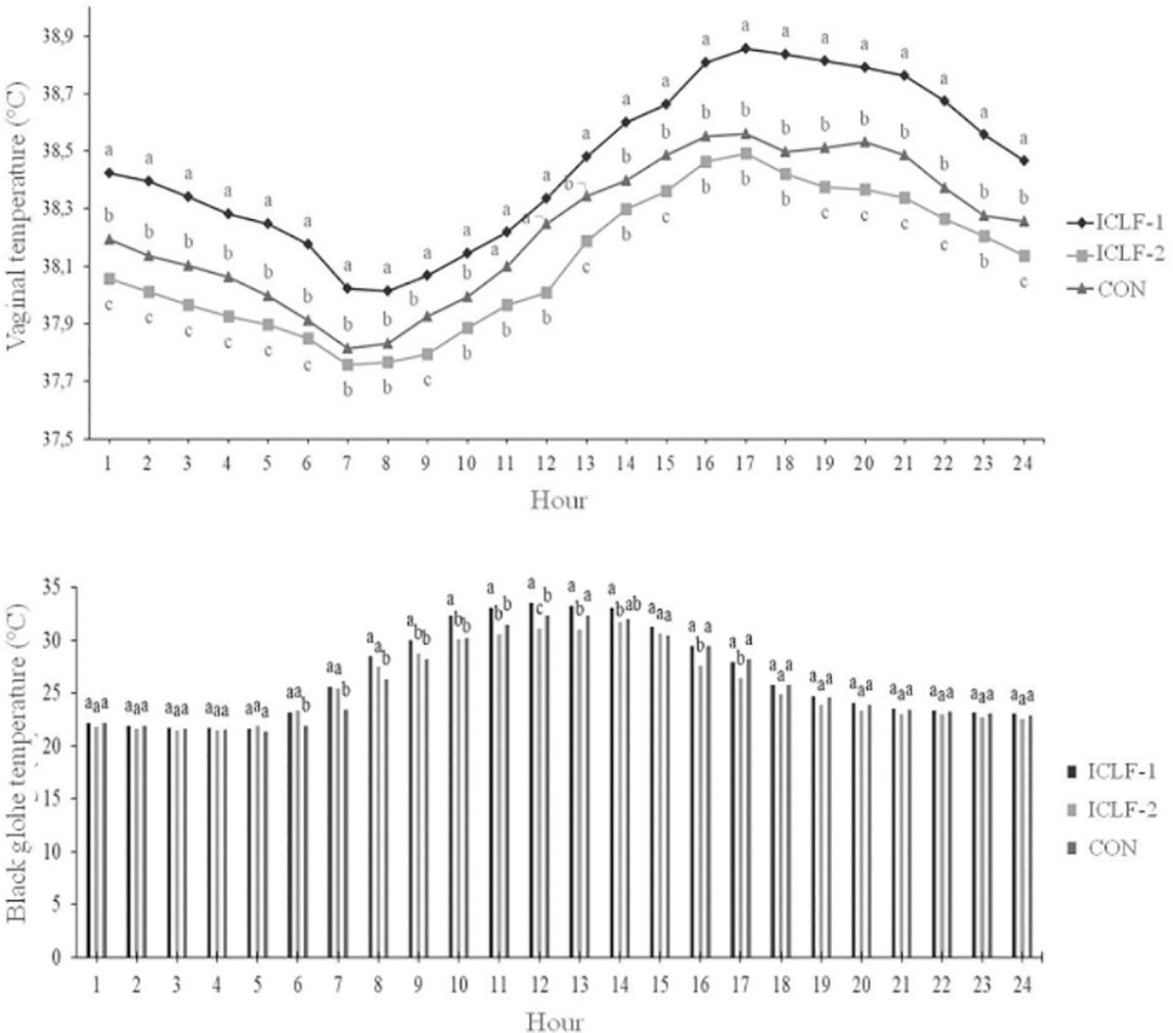
Average values of the system x time interaction of vaginal temperature measured in Nellore heifers, and the black globe temperature in integrated crop-livestock-forest (ICLF-1 and ICLF- 2) and control (CON) system. Bars with different letters between systems differ (*P* < 0.05) by the Tukey test.

Vaginal and the average black globe temperatures were different among systems and hours of the day (Fig 6). Vaginal temperature was different among systems at the period between 19:00 and 06:00, and at 09:00, 13:00 and 15:00. Vaginal temperature of animals kept on ICLF-1 was higher than ICLF-2 in all hours of the day. At 11:00 and 12:00, the CON system was similar to ICLF-1, and at 07:00, 08:00, 10:00, 14:00h and between 16:00 and 18:00, similar to ICLF-2.

For black globe temperature, we observed that systems were not different between 18:00 and 05:00. The ICLF-1 and ICLF-2 had similar black globe temperature at 06:00, 07:00 and 08:00, and CON had the least values. From 09:00 until 11:00, the black globe temperature measured on CON did not differ from ICLF-2. At 16:00 and 17:00, the black globe temperature values measured on ICLF-1 did not differ from those measured on CON. At 12:00, at the maximum peak, the black globe temperature was different among systems. No system x season interaction effect was observed for vaginal temperature, and the average value for this parameter was 38.2°C.

## Discussion

The positive correlation found between vaginal temperature and animal performance variables demonstrates an increase in BW gain as the vaginal temperature increases. We expected that this relation was the inverse, but we can suggest that animals were efficient in circumvent heat stress conditions without impairing their performance.

The positive correlation between BGHI and vaginal temperature occurred as expected since most of the climatic elements are utilized to calculate that index, which also had a positive correlation with vaginal temperature, highlighting the strong correlation observed with black globe temperature. The majority of researches relate rectal temperature with temperature humidity index (THI). For instance, Hoope et al. [16] reported negative correlation between vaginal temperature in Nellore animals and THI. The results reported by these authors contradict those found in the present study and can be explained by the use of an index that does not take into account the important effect of solar radiation. In fact, when considering solar radiation to classify how much the environment is thermally comfortable or not, this relationship can become positive. This happens because increases in this variable lead to the increase of air temperature, especially the black globe temperature, which, consequently, provides a higher heat load to the animal.

In the regression analysis, the results obtained showing that the black globe temperature explains most of the variation in the vaginal temperature may be associated to the capacity of the black globe temperature to provide information on the combined effects of air temperature, solar radiation and wind speed [17]. In fact, this result is of great interest, since the activation of the thermoregulatory mechanisms in bovines is associated with thermal environmental conditions, that is, the combination of the climatic elements and not with the effect of these elements individually.

Although the vaginal and the black globe temperatures had the same pattern of variation throughout the day, a delay in the increase of vaginal temperature occurred in relation to the black globe temperature. In the moment when black globe temperature began to increase, the vaginal temperature took, on average, two hours to increase during the winter, and one hour during the summer. When the maximum peak of black globe was reached, the vaginal temperature response took even more time, with maximum vaginal values observed two to four hours after. This delay can be explained by the existence of thermal gradient between the animal and the environment. In summary, as the ambient temperature increases, vaginal temperature is impaired after a certain period of time. Likewise, as the ambient becomes cooler, the loss of body heat also requires a period to respond physiologically, based on the reduction of the vaginal temperature values. This demonstrates that vaginal temperature is not immediately affected by the black globe temperature variation, and a period is necessary to observe changes in the animal.

The thermal comfort zone for *Bos indicus* animals is between 10 to 27°C, and the critical limit is above 35°C [18]. In the experimental conditions of the present study, the animals were under thermal discomfort in the summer, with values exceeding the zone of thermoneutrality for air temperature, and with black globe temperature above 23°C [19]. However, it is also noteworthy that, in the summer, maximum air temperature values of 41.8, 40.6 and 40.0°C were obtained in ICLF-1, ICLF-2, and CON, respectively, characterizing a condition of heat stress between 11:00 and 13:00. The relative air humidity was within the ideal range (50–70%) for cattle (50 to 70%; [20]).

The rectal temperature of Nellore animals was within the range between 37.5 and 39.5°C [21, 22]. A positive relationship between vaginal and rectal temperatures in Nellore cows was previously reported, with a difference between these temperatures of 1.1°C in the morning and 0.5°C in the afternoon [16]. Based on this assumption, despite the adverse microclimatic conditions, all heifers were able to maintain the rectal temperature within the expected range for Nellore animals.

The results of the system x year interaction demonstrate that the ICLF-1 system provided a more unfavorable thermal condition than the other evaluated systems. These results were also reflected in the vaginal temperature; heifers reared in that system presented greater increment in vaginal temperature, compared with other systems. This demonstrates that the effect of shading is not always accompanied by good thermal comfort results. The fact that the ICLF-1 has the greatest tree density is the most probable cause of vaginal and black globe temperatures were higher, because the site probably becomes more muffled, mainly by retaining more heat. The CON system had intermediate results compared to the other systems because of the greater radiation rate incident on the animal. This more favorable condition observed in the CON system can be also associated to heat loss through wind currents by the convection mechanism. In a study performed by Karvatte et al. [23] at the same experimental site, higher wind speed values were observed in the CON, followed by ICLF-2 and ICLF-1. This demonstrates that trees act as a barrier against wind currents, and the denser the system, fewer circulating winds which corroborates with the results obtained herein. Therefore, the ICLF-2 stood out compared with other systems, demonstrating that the balance between the shade availability and the microclimate pattern of this system was able to reduce the heat gain of the animals through the environment.

## Conclusions

The microclimatic conditions found in the systems, resulting from the various tree densities, modify the vaginal temperature in different degrees, demonstrating that the shading effect is not always accompanied by improvements in thermal comfort. The system with intermediate density showed a better microclimatic condition and, consequently, a lower increase in vaginal temperature. The interaction between the microclimatic variables air temperature, humidity and solar radiation resulted in adverse environmental conditions, however, Nellore heifers showed good adaptation to the environment. In conclusion, vaginal temperature is a good indicator to evaluate the thermoregulatory response in Nellore heifers.

## Supporting information

S1 Dataset Raw data collected throughout the experiment. (XLSX)
